# Chromosome-scale genome of Indian rosewood (*Dalbergia sissoo*)

**DOI:** 10.3389/fpls.2023.1218515

**Published:** 2023-08-17

**Authors:** Sunil Kumar Sahu, Min Liu, Ruirui Li, Yewen Chen, Guanlong Wang, Dongming Fang, Durgesh Nandini Sahu, Jinpu Wei, Sibo Wang, Huan Liu, Chengzhong He

**Affiliations:** ^1^ State Key Laboratory of Agricultural Genomics, Key Laboratory of Genomics, Ministry of Agriculture, BGI Research, Shenzhen, China; ^2^ BGI Life Science Joint Research Center, Northeast Forestry University, Harbin, China; ^3^ College of Life Sciences, Chongqing Normal University, Chongqing, China; ^4^ College of Science, South China Agricultural University, Guangzhou, China; ^5^ Key Laboratory for Forest Genetic & Tree Improvement and Propagation in Universities of Yunnan Province, Southwest Forestry University, Kunming, China

**Keywords:** genome, chromosome, *Dalbergia*, timber tree, comparative genomics, rosewood, phylogeny

## Introduction

*Dalbergia sissoo* (Leguminosae) is a large, deciduous tree native to the Indian subcontinent and southern Iran, and is widely distributed in the tropics (Bhagwat et al., 2015), typically reaching heights of 15-25 meters, with a maximum height of up to 30 meters (Al-Snafi, 2017). It is also known as North Indian rosewood or sheesham. It has a long, straight trunk and a dense, rounded crown. The leaves are compound, with 5-9 leaflets. The flowers are small and white or pink. The fruit is a pod that contains 1-2 seeds. *D. sissoo* is an economically important timber plant and a very useful multipurpose tree, with high-value wood that is hard, heavy, strong, durable, elastic, weather-resistant, and rot-resistant. It is also a valuable medicinal herb and high-grade spice (Son and Manh Ha, 2022). Many *Dalbergia* species are currently receiving international attention for conservation due to the overexploitation of their valuable heartwood (Hung et al., 2020). It is listed as a Least Concern species on the International Union for Conservation of Nature (IUCN) Red List and is protected by the Convention on International Trade in Endangered Species of Wild Fauna and Flora (CITES). A metabolic pathway enrichment analysis of *D. sissoo* stem extracts has shown that the differential metabolites are mainly enriched in three metabolic pathways: flavonoid biosynthesis, isoflavonoid biosynthesis, and flavonol and flavone biosynthesis. These pathways are involved in the production of flavonoids, which are a group of plant compounds with a wide range of biological activities, including antioxidant, anti-inflammatory, and antimicrobial properties. The formation and accumulation of heartwood components have always been a hot topic in the study of heartwood formation. Most of these heartwood components are extracts or secretions produced from metabolism and degradation of certain tissues. They give the heartwood a unique appearance and exhibit resistance to pathogenic fungi as potential biological protectants (Li et al., 2022). However, there is little overall understanding of the molecular basis of *D. sissoo* secondary metabolite biosynthesis pathways, which hinders progress in molecular breeding and revealing its heartwood formation mechanism. It’s also worth mentioning that research in forest tree genomics has made significant progress over the past two decades, which has provided more information to understand the genetic basis of traits such as wood formation, growth and the role of secondary metabolites (Isabel et al., 2020; Sahu et al., 2023).

Genome sequences of many woody plants have been published, such as *Juglans regia* (Marrano et al., 2020)*, Liriodendron chinense* (Chen et al., 2019)*, Eucalyptus grandis* (Myburg et al., 2014), *Dalbergia odorifera* (Hong et al., 2020), *Dipterocarpus turbinatus* (Wang et al., 2022), *Tectona grandis* (Yasodha et al., 2018), *Populus trichocarpa* (Djerbi et al., 2005) and *Picea abies* (Nystedt et al., 2013), providing the basis for further research into the molecular mechanisms regulating wood development and quality (Sahu and Liu, 2023). In this study, we successfully assembled a 661.00 Mb *D. sissoo* genome based on 214.42 Gb of 10x Genomics next-generation sequencing data, with a scaffold N50 of 7.17 Mb. Subsequently, by combining 139.93 Gb of Hi-C data, 99.9% of the Scaffolds were anchored to 10 pseudochromosomes. Chromosome-scale genome assembly will promote our understanding of the fast-growing characteristics and evolution of *D. sissoo* and facilitate the revelation of its molecular breeding and wood formation mechanisms.

## Results

### Genome assembly

**Figure 1:**
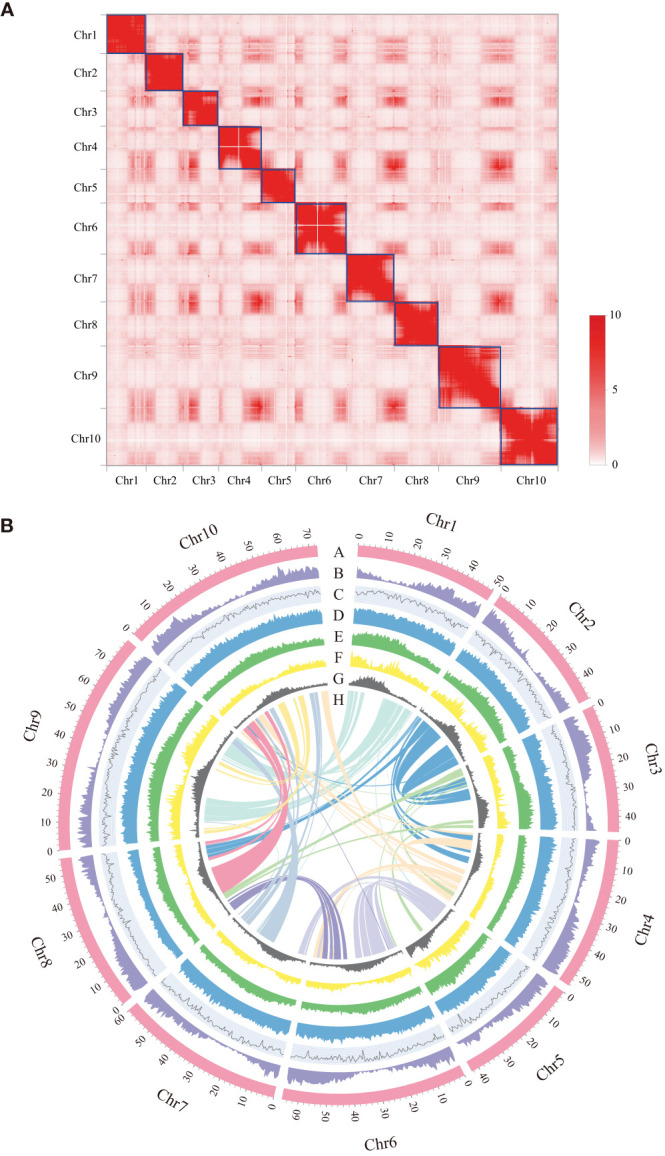
Features of *Dalbergia sissoo* genome. **(A)** Hi-C map of the *D sissoo* genome showing genome-wide all-by-all interactions. The map shows a high resolution of individual chromosomes that are scaffolded and assembled independently. The heat map colors ranging from light pink to dark red indicate the frequency of Hi-C interaction links from low to high (0–10). **(B)** Circos plot of *D sissoo* genome. Concentric circles from outermost to innermost, show (A) chromosomes and megabase values, (B) gene density, (C) GC content, (D) repeat density, (E) LTR density, (F) LTR *Copia* density, (G) LTR *Gypsy* density and (H) inter-chromosomal synteny (feature B-G are calculated in non-overlapping 500 Kb sliding windows).

To assemble the chromosome-level genome of *D. sissoo*, we generated 214.42 Gb of 10x Genomics sequencing data and 139.93 Gb of high-throughput chromatin conformation capture (Hi-C) sequencing data (Supplementary **Table S1**). The estimated genome size based on GCE (genomic charactor estimator) analysis was 756.56 Mb. The heterozygosity was 0.68%, indicating a moderately high level of heterozygosity, and the repeat content was estimated to be 48.06% (**Supplementary Table S2**), suggesting that the *D. sissoo* genome may contain a large number of repeat sequences. Finally, Supernova (v2.1.1) successfully assembled the *D. sissoo* genome into a 661.00 Mb draft, with scaffold N50 size of 7.17 Mb. Subsequently, by combining 139.93 Gb of Hi-C data, 96.6% of scaffolds were anchored to 10 pseudochromosomes (**Figure 1A**), resulting in a chromosome-level genome size of 660.37 Mb, with a scaffold N50 of 56.15 Mb (**Supplementary Table S3**). BUSCO evaluation showed 96.6% completeness, with 1329 genes aligned out of 1375 core genes, including 1282 complete single-copy BUSCOs and 47 complete duplicated BUSCOs. The BUSCO evaluation for the chromosome-level genome was 96.2% (**Supplementary Table S4-1**). DNA data alignment to the genome showed an alignment rate of 98.54%, and the GC content of the genome was 33.59% (**Table 1**), within the normal range of plant genome GC content, ruling out the possibility of bacterial contamination. Therefore, the assembled *D. sissoo* genome has high BUSCO completeness, high data alignment rate, and a normal GC distribution range, indicating a high-quality and contiguity (**Figure 1B**).

**Table 1:** Statistics of *D. sissoo* genome assembly and assessment.

Assembly		*Dalbergia sissoo*
Genome-sequencing depth(X)	10X genomics sequencing (Gb)	214.42
	Hi-C (Gb)	139.93
Estimated genome size (Mb)		756.56
Estimated heterozygosity (%)		0.68
Assembly size (Mb)		661.00
GC content (%)		33.59
Scaffold N50 (Kb)		7165.92
BUSCO completeness of assembly (%)		96.6
Complete single-copy BUSCO (%)		93.2
Complete duplicated BUSCO (%)		3.4
Total length of pseudochromosome assembly (Mb)		660.37
Pseudochromosome number		10
Scaffold N50 of pseudochromosome assembly (Kb)		56151.84
BUSCO completeness of pseudochromosome assembly (%)		96.2
The rate of pseudochromosome anchored genome (%)		99.9

### Genome annotation

Based on *de novo* prediction and comparison with known repeat databases, repeat sequences accounted for 52.8% of the genome, with LTR types being the most common (**Supplementary Table S5**). Structural annotation identified 29,737 protein-coding genes in the genome, with a BUSCO evaluation result of 95.40% (**Supplementary Table S6**), indicating high completeness of the predicted gene set. The functional annotation revealed that 28,689 genes had functional information, which represents 96.48% (**Supplementary Table S7**). In addition, noncoding RNA in the *D. sissoo* genome was predicted, and a total of 3,848 ncRNAs were identified, with a sequence length of 0.48 Mb, accounting for 0.07% of the genome. Among them, 123 miRNAs, 1,141 tRNAs, 1,812 rRNAs, and 770 snRNAs were identified (**Supplementary Table S8**, **Table 2**).

**Table 2:** Genome annotation of *D. sissoo*.

Annotation	*Dalbergia sissoo*
Number of predicted protein-coding genes	29740
Average gene length (bp)	4135.19
Average exon length (bp)	222.06
Average exon number per gene	5.47
Average intron length (bp)	654.18
miRNAs	123
rRNAs	906
tRNAs	1141
Percentage of repeat sequencing (%)	52.8
LTR *Copia* (%)	8.92
LTR *Gypsy* (%)	27.63
LINE (%)	1.29
SINE (%)	0.02
DNA transposons (%)	7.84
Percentage of functional annotation genes (%)	96.48

### Phylogenetic analysis and species divergence time estimation

**Figure 2:**
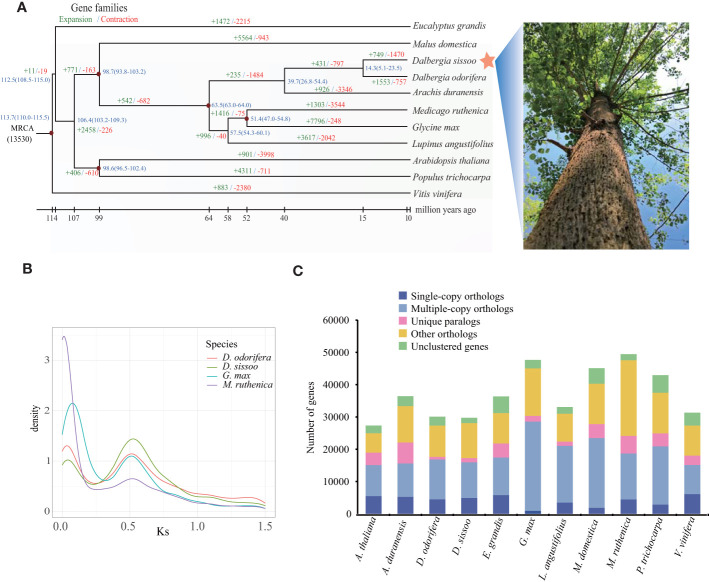
Evolution of the *D sissoo* genome. **(A)** Phylogenetic tree showing the taxonomic position of *D. sissoo*. The blue numbers denote divergence time of each node (MYA: million years ago). The numbers in green indicate the number of gene families that expanded in the species during evolution, and the numbers in red indicate the number of gene families that contracted. **(B)** Ks distribution plot. **(C)** The distribution of single-copy, multiple-copy, unique, and other genes in the 11 plant species.

To determine the evolutionary relationships between *D. sissoo* and other species, we identified 108 single-copy orthologs using OrthoFinder (v2.3.1) from 11 representative plant species (**Supplementary Table S9**). The protein sequence alignment of these single-copy orthologs was clustered using the MCL algorithm (v14-137) included in OrthoFinder and used to generate a phylogenetic tree with *Vitis vinifera* as the outgroup. The phylogenetic tree showed that *D. sissoo* first diverged from the common ancestors of *Arabidopsis thaliana* and *P. trichocarpa*, and then later from *Malus domestica*. The closest evolutionary relationship of *D. sissoo* was with *D. odorifera*, estimated to have diverged about 14.3 million years ago (MYA) (**Figure 2A**).

### Gene family expansion and contraction

Gene family evolution analysis using CAFE on the 11 plant species showed that *D. sissoo* had 749 significantly expanded and 1470 significantly contracted gene families. KEGG enrichment analysis of the expanded gene families revealed that most of the genes were related to secondary metabolites such as flavonoids, terpenoids, and ABC transporters. In addition, genes related to photosynthesis were also expanded significantly (**Supplementary Figure 3A**). The GO enrichment analysis of the expansion gene family of *D. sissoo* also enriched many photosynthesis-related terms such as photosynthesis, light reaction, photosystem I, photosystem II reaction center, and chlorophyll II binding, and so on (**Supplementary Figure 3B**), suggesting that the fast-growing characteristics of *D. sissoo* are the result of the expansion of a large number of photosynthesis-related genes. The contracted gene families were significantly enriched in 10 KEGG pathways, including vitamin B6 metabolism, phenylpropanoid biosynthesis, cutin, suberin and wax biosynthesis, and the citric acid cycle. (**Supplementary Figure 3C**).

### Whole-genome duplication analysis

Here, we employed wgd software and compared the relative genomic CDS sequences of *D. odorifera*, *Glycine Max* (Schmutz et al., 2010) and *Medicago ruthenica* (Yin et al., 2021) to calculate Ks values, and the occurrence of whole genome duplication (WGD) events was judged by the distribution of Ks values (**Figure 2B**). It was found that *D. sissoo*, *D. odorifera*, *G. Max* and *M. ruthenica* experienced a WGD event common to legumes, from which we inferred that the WGD event of *D. sissoo* occurred close to the first WGD event of *G. Max* (~59 Mya) (Schmutz et al., 2010), followed by another independent WGD event of the genus *Glycine* in which soybean is located ~13 Mya. No independent WGD event was observed for the genus *Dalbergia*. The distribution of four gene types - single-copy, multiple-copy, unique, and other genes - across various tree species are displayed in **Figure 2C**.

### Identification of gene duplication types

The number of genes in the *D. sissoo* genome for whole genome duplication (WGD), tandem duplication (TD), proximal duplication (PD), and transposition duplication (TRD), and dispersed duplication (DD) types were 6522, 800, 607, 2895 and 4500, respectively. The predicted duplication types were compared with the expanded gene family, and five replication types had 578, 223, 183, 311, and 734 genes in the expanded gene family, totaling 2029, respectively (**Supplementary Figure 2A**). Among all the WGD and DD type genes contributed the most to the expansion of the *D. sissoo* gene family.

## Discussion

*Dalbergia sissoo*, commonly known as Indian rosewood, is an ecologically and economically vital tropical timber species (Bhagwat et al., 2015). However, a lack of genomic resources has constrained genetic research in *D. sissoo*, including studies of heartwood formation, a key trait for timber quality (Hong et al., 2020; Hung et al., 2020) (Li et al., 2022). The availability of high-quality chromosome-level reference genomes can greatly advance genetic research in economically valuable plant species (Sahu and Liu, 2023). In this study, the successful assembly of a chromosome-scale *D. sissoo* genome represents a major advance for investigating heartwood formation and highlights the potential impact on conserving and sustainably utilizing this important tree. The *D. sissoo* genome revealed typical features of woody plants, with relatively few genes (29,737) compared to herbaceous species but abundant repeats (52%) and secondary metabolites (Hong et al., 2020) (Neale and Kremer, 2011; Yasodha et al., 2018). Comparative genomics showed expansions in several gene families related to heartwood synthesis and defense compared to other trees (Sahu et al., 2019; Fan et al., 2020). These expanded genes likely contribute to *D. sissoo*’s production of medicinal chemicals and durable, insect-resistant timber. These findings mirror observations in other tropical genera like Eucalyptus and Mango (Myburg et al., 2014). The high-quality reference genome will facilitate identifying genes and networks involved in heartwood formation and valuable wood traits, providing useful markers for breeding and conservation (Yasodha et al., 2018; Hung et al., 2020; Guo et al., 2021). It also enables population genomics and association studies to elucidate adaptations in these ecologically and economically vital Neotropical trees (Naidoo et al., 2019; Hong et al., 2020). Furthermore, the genome can serve as a reference for resequencing other threatened *Dalbergia* species exploited for timber. The *D. sissoo* draft genome thus represents a vital contribution for genetics-based improvement and sustainable forestry of rosewoods and tropical trees.

## Materials and methods

### Plant materials, library construction, and sequencing

The fresh material of *D. sissoo* was obtained from Ruili Botanical Garden in Yunnan Province, China. The total DNA of young leaves was extracted by the standard (cetyltrimethylammonium bromide) CTAB method (Sahu et al., 2012), and the library was constructed according to the standard procedure of 10X genomics library kit (Chromium Genome Chip Kit v1, 10X Genomics, Pleasanton, USA), and the obtained target DNA library was sequenced using BGISEQ-500 sequencer to obtain read length data. The RNA was extracted from young leaves, xylem and phloem tissues using the PureLink RNA Mini Kit (Thermo Fisher Scientific, Carlsbad, CA, USA), and the library was built according to the standard procedure of the TruSeq RNA Sample Preparation Kit manual (Illumina, San Diego, CA, USA), and the obtained library was sequenced using the BGISEQ-500 platform to obtain transcriptome data at 100bp pair-end. Furthermore, according to the standard procedure of the Hi-C experiment, young leaves of *D. sissoo* were selected for cross-linked DNA extraction and library construction, and the constructed Hi-C library was sequenced on the BGISEQ-500 platform for 100bp pair-end sequencing to obtain Hi-C data. DNA and RNA data were subjected to FastQC (v 0.11.9) analysis to assess the quality of the raw downstream data, followed by filtering using Trimmomatic (v 0.39) (Bolger et al., 2014) based on the QC results, and the statistical information of each filtered data is shown in **Supplementary Table 1**.

### Genome assembly and assessment

We used DNA data from 10X genomics an input for the GCE (v 1.0.0) (Liu et al., 2013) and Kmerfreq_16bit (Wang et al., 2020) for genome size prediction. The genome was then preliminarily assembled using the supernova software for the 10X genomics data. To further improve the assembly quality and construct the genome at the chromosome level, the anchoring of the chromosome was performed using Juicer (v 1.5) (Durand et al., 2016) and 3D-DNA (v180419) (Dudchenko et al., 2017).

After obtaining the results of the genome assembly, the quality of the genome assembly was assessed in five main ways: (1) Counting Scaffold N50 and N90 length. (2) Using BUSCO (v3.0.1) (Simão et al., 2015) to assess the number and proportion of results of chromosome assembly occupying the angiosperm core gene set database (embryophyta_odb10). (3) All filtered DNA data were compared back to the genome using Bwa-mem (v0.7.12)(Li, 2013), and the read comparison rate was counted. (4) All filtered RNA data were compared back to the genome using Tophat2 (v2.1.1) (Kim et al., 2013), and the read ratio was counted. (5) In order to verify the gene contamination of the assembly, the above comparison results were used as input in soap.coverage 2.27 (http://soap.genomics.org.cn/) to calculate the relationship between GC content and coverage, and to display the distribution between the coverage of reads and GC content.

### Genome annotation

Genome annotation mainly includes repetitive sequence annotation, gene annotation and non-coding RNA annotation. Firstly, repeat annotation was performed using a method based on *ab initio* prediction and comparison with a database of known repeats. For tandem repeat sequences, tandem structure identification was performed using TRF (v 4.10.0) (Benson, 1999). For dispersed repeat sequences, LTR_Finder (v 1.0.7) (Xu and Wang, 2007) was first used for *de novo* prediction of LTR sequences based on repetitive features, and RepeatModeler (v 2.0.1) (Flynn et al., 2020) was used for *ab initio* prediction of other repetitive elements based on the comparison of the sequences themselves, and a local repeat database was constructed. Finally, by combining the local repeat database and the downloaded repeat sequence library, Repbase (v 21.12) (Jurka, 2000), using RepeatMasker (v 4.0.7) (Chen, 2004) and RepeatProteinMask (v 4.0.7) (Chen, 2004) to perform the prediction at the nucleic acid and protein sequencing levels, respectively.

For gene structure annotation, we adopted a combination of *de novo*, homology, and transcriptome-based annotations. For *de novo* annotation, the transcriptome reads were first compared back to the reference genome using Hisat2 (v 2.2.1)(Kim et al., 2015), and the correctly compared reads were assembled using Stringtie (v 1.3.3b) (Pertea et al., 2015) and then the obtained transcripts were compared back to the reference genome by PASA (v 2.1.0) (Haas et al., 2008) for gene model prediction. For homology annotations, gene sets of the leguminous relatives of *Arachis duranensis*, *G. max*, and *M. truncatula*), which are also in the same legume family as *D. sissoo*, were downloaded from the NCBI or Phytozome websites as evidence of homology annotation. For transcriptome annotation, transcriptome data were assembled using Trinity (v 2.0.6) (Haas et al., 2013), and the transcripts obtained were used as transcriptome annotation evidence. Finally, the above evidence obtained after *de novo* annotation, homology annotation, and transcriptome annotation were jointly integrated as input files for MAKER (v 2.31.11) (Cantarel et al., 2008), which was run to output the final gene structure annotation results. The protein sequences of 29737 genes obtained from structural annotation were compared with Interpro, Swissprot, KEGG, NR, Trembl, and COG functional databases using BLASTP (v2.2.31) (Camacho et al., 2009) with the threshold set to E-value <le-05. For ncRNA annotation, BLASTN (v 2.2.26)(Camacho et al., 2009) was used in combination with the highly conserved rRNA database of closely related species to search for rRNAs; tRNAscan-SE (v 1.3.1) (Lowe and Eddy, 1997) was used to search for genomic tRNAs based on secondary structure features; infernal (v 1.1.2) (Nawrocki and Eddy, 2013) was used in combination with the Rfam database (v 12.0) (Nawrocki et al., 2015) for secondary structure detection of snRNAs and microRNAs.

### Phylogenomic analysis

Gene family clustering analysis was performed using OrthoFinder (v2.3.1) (Emms and Kelly, 2015) combining protein sequences of genes encoding a total of 10 plants: *A. thaliana*, *A. duranensis*, *P. trichocarpa*, *D. odorifera*, *Lupinus angustifolius*, *G. max*, *E. grandis*, *M. ruthenica*, *Malus domestica*, and *V. vinifera*. Based on OrthoFinder clustering results, single-copy orthologous gene families were extracted using Linux commands, and then all protein sequences of single-copy gene families were compared in multiple sequences using MAFFT (v7.310) (Katoh and Standley, 2013). After filtering, a sequence supermatrix was generated by concatenating all genes in the single-copy gene family in order of species origin. Finally, this matrix file was used as the input of IQTREE (v1.6.1) (Nguyen et al., 2015) for phylogenetic tree construction. The obtained tree construction results were used to root and visualize the phylogenetic tree using FigTree (v1.4) (http://tree.bio.ed.ac.uk/software/figtree/). Subsequently, the phylogenetic tree was further combined with the MCMCTREE module in the PAML (v4.5) (Yang, 2007) and the TimeTree divergence time database (http://www.timetree.org) information to perform divergence time estimation of target species in the tree.

### Gene family analysis

The expansion and contraction analysis of the gene family was performed using CAFÉ (v4.2.1) (De Bie et al., 2006). Firstly, the species gene family number statistics file obtained from OrthoFinder clustering and the MCMCTREE predicted species divergence time tree file were used as input to CAFÉ. CAFÉ employs the self-contained birth-and-death evolution model to compare the number of genes of different species in each gene family with each other to derive the expansion and contraction of gene families, followed by enrichment analysis based on GO and KEGG annotations to determine the functional meaning of the expanded and contracted genes.

### Whole-genome duplication analysis

We used the wgd package’s BLASTP module (Zwaenepoel and Van de Peer, 2019) to conduct self-comparison and inter-comparison of CDS sequences of *D. sissoo* and its close relatives, and MCL clustering was used to find direct homologous and paralogous gene families based on BLASTP results; then MAFFT module was used to perform multiple sequence concatenation for each gene family, the Codeml module was used to calculate Ks, and finally the R scripts were used to derive Ks distribution maps of Ks of different species.

### Identification of gene duplication events

DupGen_finder (Qiao et al., 2019) was used to identify the gene duplication type of *D. sissoo* genome. First, BLASTP (v2.2.26) was used to compare the gene and protein sequences of *D. sissoo* (e<10-5). Then gene position information was extracted from the gene structure annotation GFF file, including the sequence ID where the gene is located, the gene ID, and the start and end position information of the gene on the sequence. Finally, the alignment result file and gene location information were used as an input in DupGen_finder.unique to predict gene duplication types.

## Data availability statement

The raw sequencing data and genome assembly data reported in this article have been deposited into CNGB Sequence Archive (CNSA) of the China National GeneBank DataBase (CNGBdb) with accession number CNP0004041 (https://db.cngb.org/search/project/CNP0004041/). Annotation files are available via figshare (https://figshare.com/articles/dataset/22662127).

## Author contributions

HL, SS, and CH led and designed this project. HL and SS conceived the study. SS, and JW collected the leaf and tissue samples. SS, ML, RL, GW, and YC contributed to the sample preparation and performed the genome and chromosome-scale assembly. SS, ML, SW, YC, DF, GW, DS, RL, and SW performed annotation and comparative genomic analyses. SS, RL, and ML wrote the original draft manuscript. SW, ML, CH, DS, DF, and HL, revised and edited the manuscript. All authors read and approved the final manuscript.
