# Supplementary material for: Chromosome-scale genome of Indian rosewood (Dalbergia sissoo)
Source: Front Plant Sci. 2023 Aug 17;14:1218515. doi: 10.3389/fpls.2023.1218515 (PMC10470032; doi:10.3389/fpls.2023.1218515)
Supplement: Supplementary file 1 [file DataSheet_1.docx]

**Supplementary figures**

**
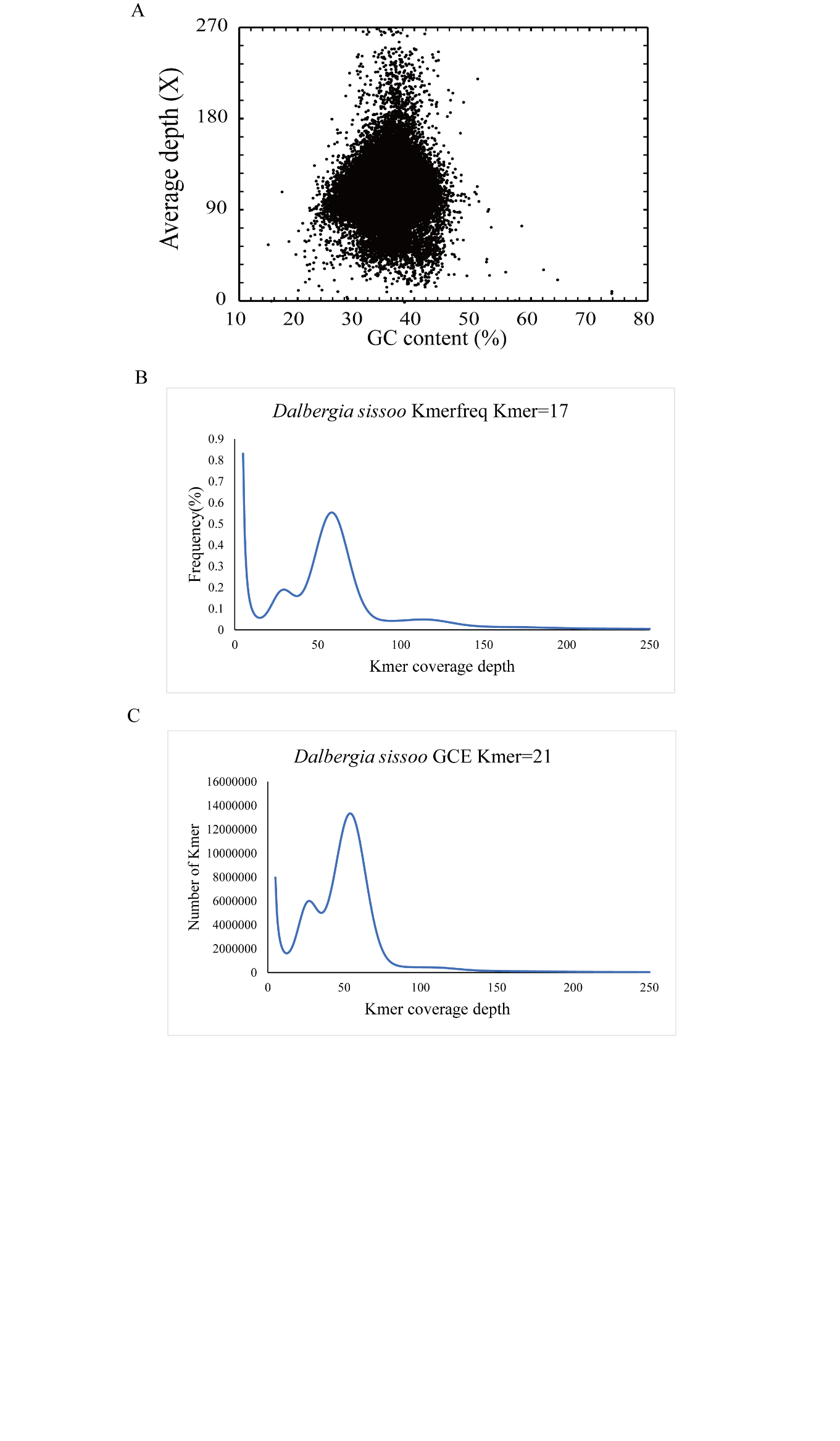
**

**Supplementary figure 1.** GC depth and Kmer distribution in *D. sissoo*. **(A)** GC depth distribution plot. **(B)**Kmer frequency distribution plot obtained by Kmerfreq. **(C)** Distribution of Kmer numbers obtained by GCE.

**
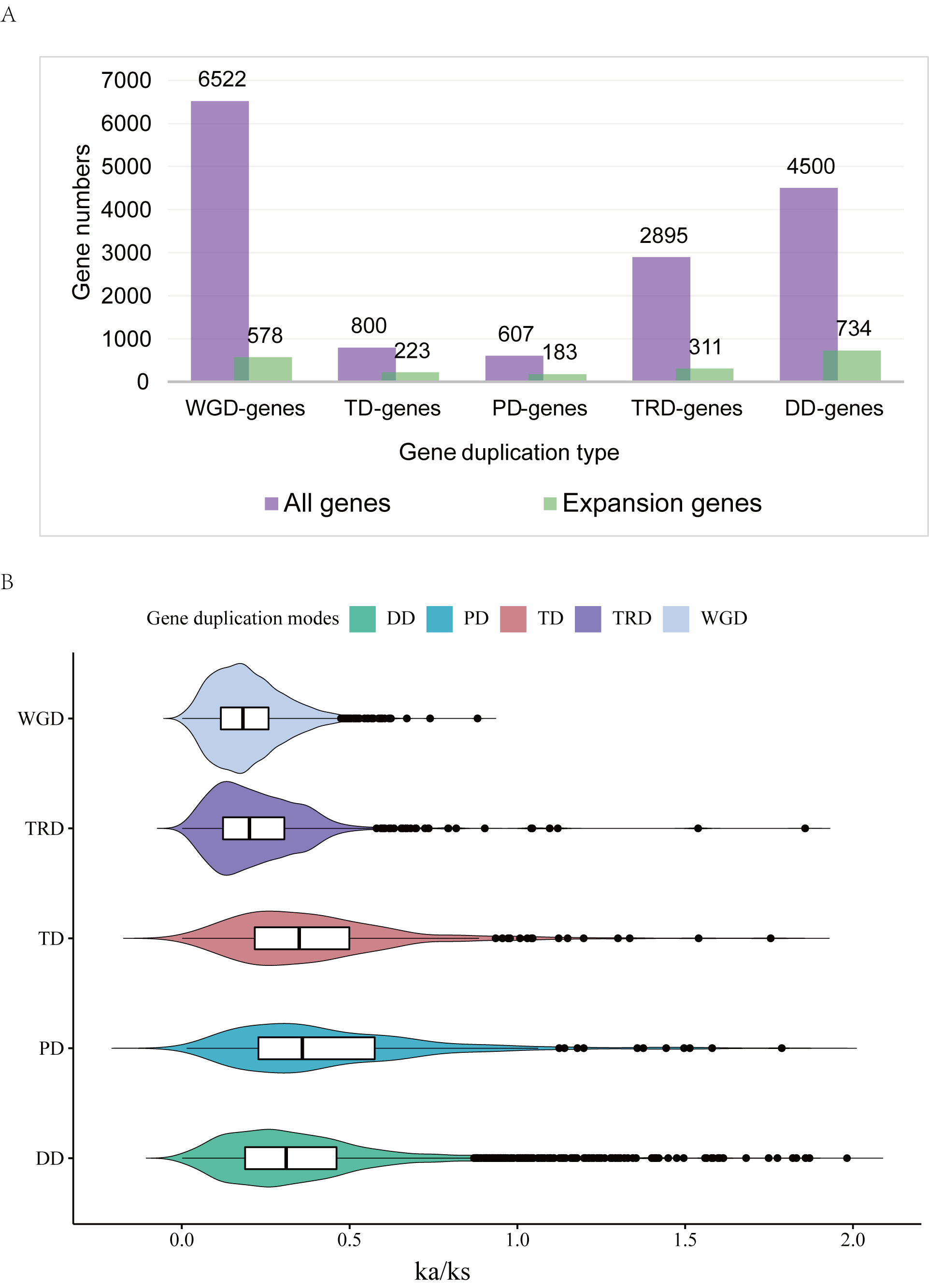
Supplementary figure 2.** Identification of gene duplication. **(A)** The distribution of five modes of duplicate genes in *D. sissoo*. **(B)** The Ka/Ks ratio distributions of gene pairs derived from five modes of duplication.

**
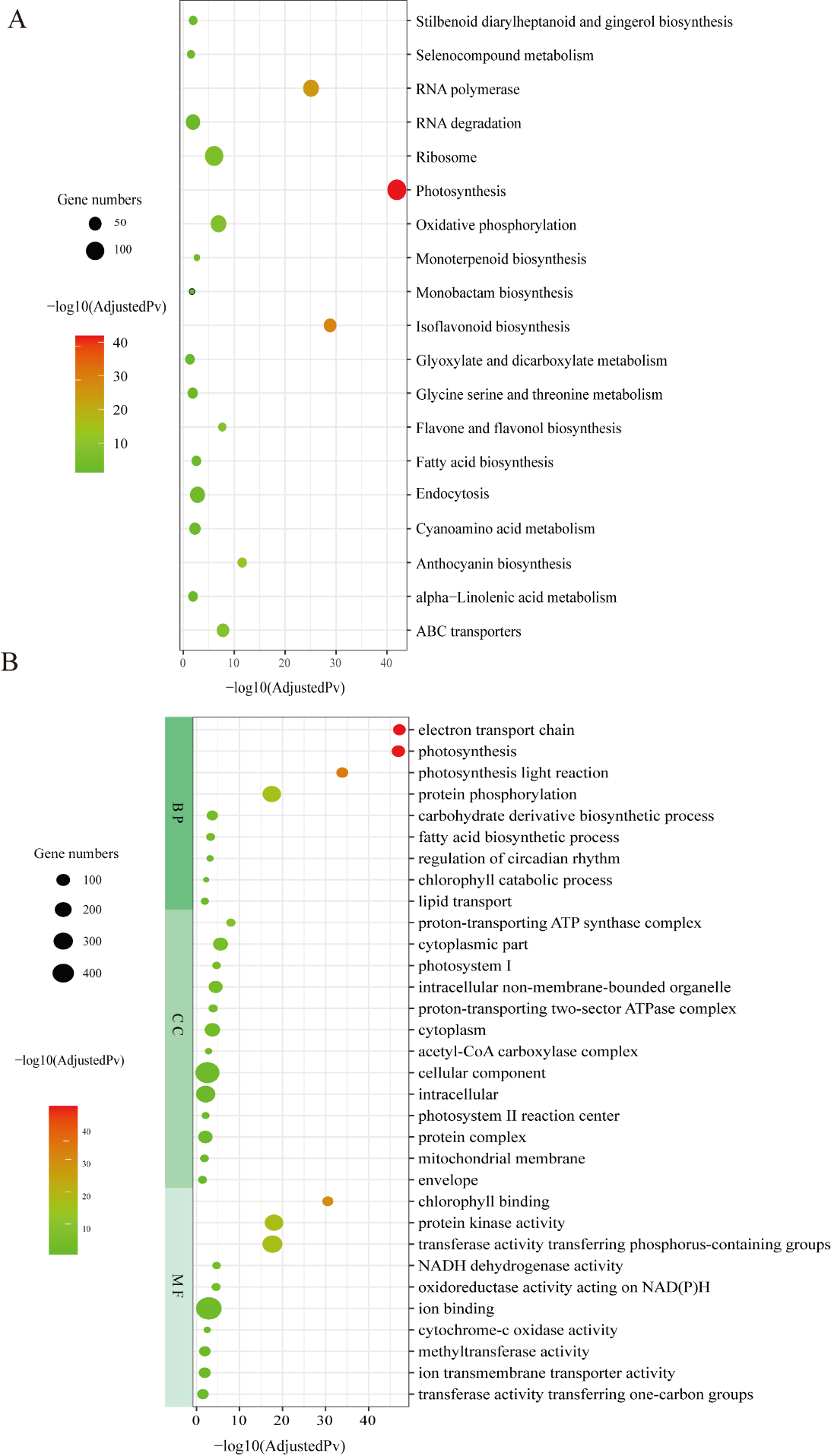
**


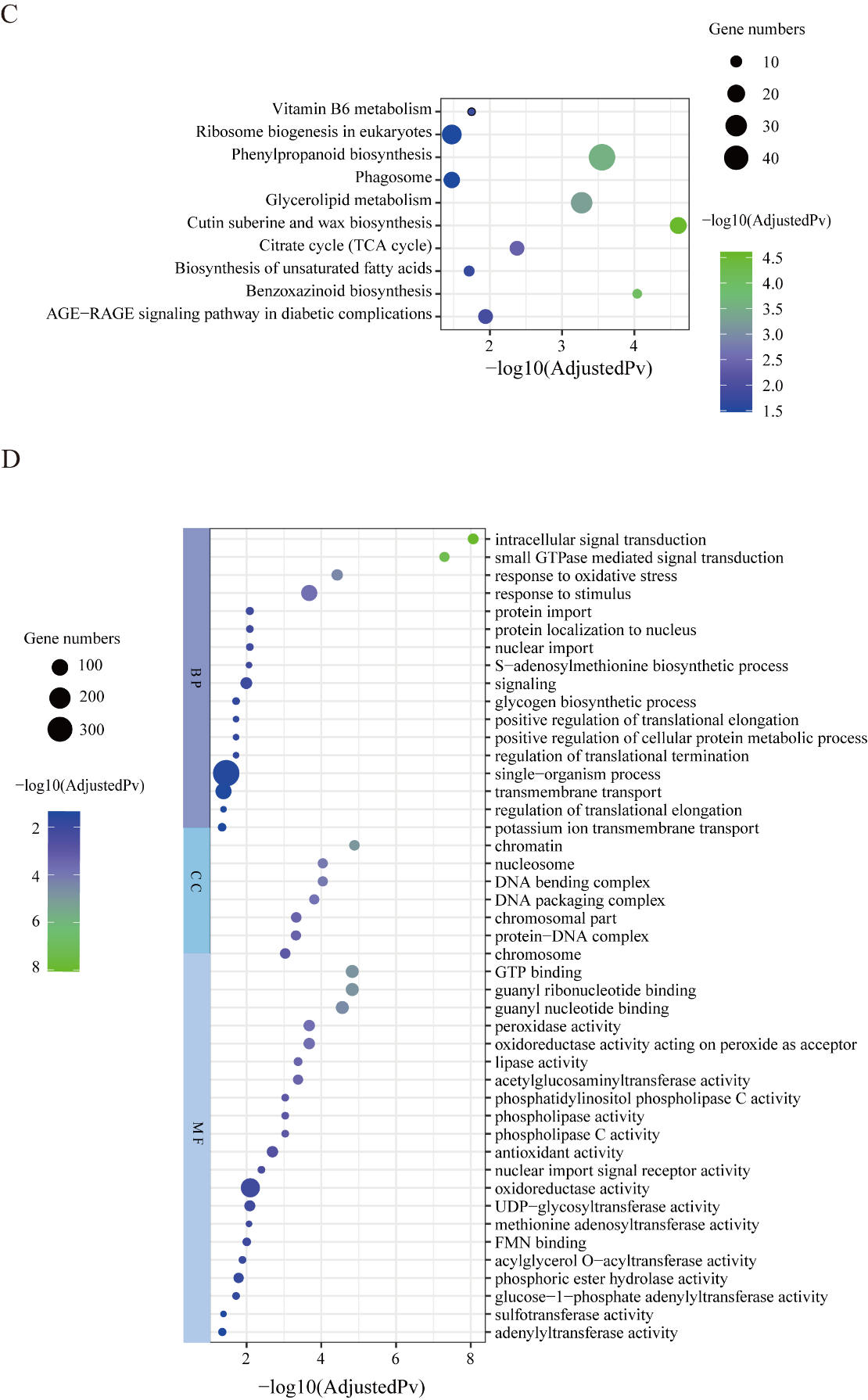
**Supplementary figure 3.** **(A)** KEGG enrichment analysis for *D. sissoo* expanded genes. **(B)** GO functional enrichment analysis for *D. sissoo* expanded genes. **(C)** KEGG enrichment analysis for *D. sissoo* contracted genes. **(D)** GO functional enrichment analysis for *D. sissoo* contracted genes. The enriched GO terms involved biological process (BP), cellular component (CC) and molecular function (MF). The color of circles represents the statistical significance of enriched GO terms. The size of the circles indicates the number of genes in a GO term. Detailed information is shown in Supplementary table S10-S13.
